# Underlying Co-Morbidity Reveals Unique Immune Signatures in Type II Diabetes Patients Infected With SARS-CoV2

**DOI:** 10.3389/fimmu.2022.848335

**Published:** 2022-04-27

**Authors:** Soumya Sengupta, Gargee Bhattacharya, Sanchari Chatterjee, Ankita Datey, Shubham K. Shaw, Sandhya Suranjika, Paritosh Nath, Prakash K. Barik, Punit Prasad, Soma Chattopadhyay, Rajeeb K. Swain, Ajay Parida, Satish Devadas

**Affiliations:** ^1^ Department of Infectious Disease Biology, Institute of Life Sciences, Bhubaneswar, India; ^2^ Regional Centre for Biotechnology (RCB), 3rd Milestone, Faridabad-Gurgaon, India; ^3^ School of Biotechnology, Kalinga Institute of Industrial Technology (KIIT) University, Bhubaneswar, India

**Keywords:** SARS-CoV2, type II diabetes, whole blood immunophenotyping, innate immune response, adaptive immune response

## Abstract

**Background:**

SARS-CoV2 infection in patients with comorbidities, particularly T2DM, has been a major challenge globally and has been shown to be associated with high morbidity and mortality. Here, we did whole blood immunophenotyping along with plasma cytokine, chemokine, antibody isotyping, and viral load from oropharyngeal swab to understand the immune pathology in the T2DM patients infected with SARS-CoV2.

**Methods:**

Blood samples from 25 Covid-19 positive patients having T2DM, 10 Covid-19 positive patients not having T2DM, and 10 Covid-19 negative, non-diabetic healthy controls were assessed for various immune cells by analyzing for their signature surface proteins in mass cytometry. Circulating cytokines, chemokines, and antibody isotypes were determined from plasma while viral copy number was determined from oropharyngeal swabs. All our representative data corroborated with laboratory findings.

**Results:**

Our observations encompass T2DM patients having elevated levels of both type I and type II cytokines and higher levels of circulating IgA, IgM, IgG1, and IgG2 as compared to NDM and healthy volunteers. They also displayed higher percentages of granulocytes, mDCs, plasmablasts, Th2-like cells, CD4^+^ EM cells, and CD8^+^ TE cells as compared to healthy volunteers. T2DM patients also displayed lower percentages of pDCs, lymphocytes, CD8^+^ TE cells, CD4^+^, and CD8^+^ EM.

**Conclusion:**

Our study demonstrated that patients with T2DM displayed higher inflammatory markers and a dysregulated anti-viral and anti-inflammatory response when compared to NDM and healthy controls and the dysregulated immune response may be attributed to meta inflammation.

## Introduction

Since the outbreak of the novel coronavirus disease (Covid-19) in late 2019, India has recorded over 34 million cases and 454,745 deaths as of October 25, 2021 and now has the second highest number of cases in the world, after the United States. Essentially, SARS-Cov2 is a betacoronavirus, belonging to the Coronaviridae family and is closely related to SARS and MERS, which were responsible for earlier disease outbreaks in 2003 and 2011, respectively (1). Broadly, Covid-19 cases were and are still classified into symptomatic and asymptomatic, based on the presence or absence of symptoms and eventual severity based on immune response and/or its failure. With respect to the symptoms of Covid-19 infection, symptomatic patients had fever, dry cough, shortness of breath, acute respiratory distress, loss of taste and smell, and in certain cases diarrhea (2, 3). Based on the intensity of these symptoms, the patients are classified into mild and severe, during diagnosis and prognosis of the severe patients is critical for recovery. However, the major challenge arises from the huge population of asymptomatic cases, as they are responsible for the undetected spread of infection.

In general, disease severity in Covid-19 is associated with lymphopenia, cytokine storm, blood coagulation, drop in pO_2_ levels, etc. (4, 5). However, with respect to the Indian population, these clinical parameters and the associated immune response were not definitely present in most Covid-19 infected patients. Interestingly, one of the major attributes of Covid-19 in the Indian population, especially in the first wave, was quick recovery, but the underlying immunological mechanism was and is yet to be understood. However, similar to the first wave, 70% of India’s mortality in Covid-19 in the second wave is still attributed to comorbidities, specifically type II diabetes (T2DM). Although vaccination drives initiated by the government aim to protect the population in general, the overwhelming number of these patients poses a major challenge to recovery and recuperation of the individual. In that respect, co-relation between comorbidities including T2DM and viral load, T2DM and glucose levels, etc. in the patient can play a significant role in dictating the immune response and eventual outcome of Covid-19 infection. Therefore, it is crucial to understand the interplay of these factors along with immunological parameters to give us a comprehensive idea about the status of T2DM patients having Covid-19.

Immune response can be divided into three types, namely, type I (antiviral), type II (anti helminthic), and type III (antifungal). Type I response mainly constitutes T-bet and Interferon gamma (IFN-γ) mediated response, which is against intracellular pathogens, including viruses. On the other hand, type II response is mediated by GATA-3 and is mainly against helminthic worms, which are carried out by effector molecules such as IL-4, IL-9, and IgE. The third type of response (type III response) is mediated by ROR-γt and effector molecules such as IL-17A, IL-17F, 1L-22, etc. are responsible for controlling fungal infections (6, 7). Longitudinal analysis has shown an immune dysregulation in Covid-19 patients and people with T2DM contracting Covid-19 infection had higher innate immune cells, lower T lymphocytes, a sustained increase in antiviral, anti-fungal response, and higher type 2 response such as IL-5, IL-13, IgE, and eosinophils (8).

Altogether, the immunological response in Covid-19 patients with co-morbidity is an evolving area of study and these patients represent higher vulnerability in comparison to the ones without any co-morbidity or other co-morbidities. Our study aims to elucidate the differences between T2DM patients having Covid-19 as compared to those devoid of T2DM, based on multiple parameters such as viral load, cytokine *milieu*, and immune cells, which will give us a comprehensive idea about the role played by T2DM in Covid-19 pathogenesis.

## Materials and Methods

### Population and Samples

A total of 25 samples with T2DM who were infected with SARS-CoV2 and 10 samples without T2DM (NDM) (non-diabetes mellitus) who were infected with SARS-CoV2 were collected from a tertiary hospital within 4 days of the patient’s admission. Type II diabetes in these patients was confirmed by previous clinical history and for this particular study was assessed and confirmed by fasting plasma glucose level and glycated hemoglobin while other clinical and biochemical parameters as requested by their consultant medical practitioner were used to assess for organ health. Among these, 22 patients with T2DM had hypertension. The T2DM or NDM patients neither reported nor were assessed for other co-morbidities during the infection. The detailed laboratory findings are given in **Table 1**. All the T2DM patients had severe symptoms, as assessed by the clinician. The NDM patients either had mild symptoms or were asymptomatic. The details of the drug administered to the patients are given in **Supplementary Table S3**. Additionally, 10 healthy volunteers who tested negative for Covid-19 were included in the study to provide basal and steady state biochemical and cytokine profile. These healthy controls (HC) did not have any systemic inflammation, chronic diseases, autoimmunity, infection, or malignancies. The details of their hematological parameters are given in **Supplementary Table S1**.

**Table 1:** Demographic details and laboratory findings of T2DM and NDM patients infected with SARS-CoV2.

	T2DM n = 25	NDM n = 10	P value
Age, median (IQR), years	46 (36-59)	49 (40.25-55.50)	N. A
Sex, man/woman, n (%)	17 (68%)/8 (32%)	7 (70%)/3 (10%)	N. A
Hemoglobin, gm/dl median (IQR)	12.2 (11.35-13.60)	14.25 (13.7-14.9)	0.0475
Platelets 10^3^/µL median (IQR)	193 (154.5-235)	165 (159.5-197.5)	0.4027
White blood cells 10^3^/µL, median (IQR)	6.1 (5.00-57.65)	5.8 (5.2-5.575)	0.4172
Neutrophils %, median (IQR)	75 (65.5-84)	55 (44.75-62)	0.0004
Lymphocytes %, median (IQR)	22 (15.5-29.75)	36 (26.75-39.75)	0.0349
Eosinophils %, median (IQR)	0	3 (3-4.5)	<0.0001
Monocytes %, median (IQR)	7 (5-9)	9 (8.5-10)	0.0373
D-dimer µg/mL, median (IQR)	0.45 (0.29-1.61)	0.19 (0.15-0.25)	0.0048
Ferritin ng/mL, median (IQR)	224.7 (145.9-464.1)	115 (110-289.5)	0.4975
Fasting plasma glucose mg/dL, median (IQR)	215 (142.72-244)	95.5 (88-101)	<0.0001
Hb1AC %, median (IQR)	6.9 (6-9)	N. T	N. A
CRP mg/mL, median (IQR)	69.62 (34.95-211)	13.28 (4.44-18.65)	0.0013
Serum SGOT U/L, median (IQR)	46.9 (30.2-64.6)	38 (30.75-43.25)	0.4432
Serum SGPT U/L, median (IQR)	27.6 (20-42)	40.5 (24.5-76.5)	0.1335

Hb1AC, glycated hemoglobin; CRP, C-reactive protein; Serum SGOT, serum glutamic-oxaloacetic transaminase; Serum SGPT, serum glutamic-pyruvic transaminase; N.T., not tested; N.A., not applicable.

Briefly, 5 ml blood was collected in BD Vacutainer EDTA tubes (BD 367863) and 270 μl of whole blood from 10 T2DM patients, 5 NDM patients, and 5 healthy volunteers was used for deep immune profiling by mass cytometry. The tubes were spun at 1600 g for 20 min at room temperature. Plasma was then collected and stored in -80°C until further analysis. The details of all the reagents and software used are given in **Supplementary Table S2**.

### SARS-CoV2 Viral Load Detection in Respiratory Specimen and Plasma

There were 300 μl each of oropharyngeal swab and plasma taken for viral RNA extraction using TAN Bead Maelstrom 4800 as per the manufacturer’s instructions. The extracted viral RNA was stored at -80°C until further use (9).

### qRT-PCR

The qRT-PCR was performed using 5 μl of the extracted RNA from samples using the TRUPCR SARS-CoV-2 RT qPCR Kit V-2.0. The human RNase P served as an internal control whereas envelope (E) and nucleocapsid (N) genes were targeted for SARS-CoV-2 amplification (9).

### Viral Copy Number Determination

The viral copy number was determined for the above-mentioned samples by generating a standard curve of SARS CoV-2 N (nucleocapsid) gene. The N gene was cloned into pBiEx vector, and 10-fold serial dilutions of the plasmid were done to obtain a standard curve. The percentage of copy number/ml was calculated from the corresponding Ct values of all the samples. For obtaining Ct values, cDNA was prepared from the extracted RNA using random hexamers by TAKARA primescript 1st strand cDNA synthesis kit (Kusatsu, Japan). The cDNA was subjected to qPCR (Mesagreen SYBR Green-No ROX, Eurogentec, Belgium) using nucleocapsid gene specific primers. (FP: GTAACACAAGCTTTCGGCAG and RP: GTGTGACTTCCATGCCAATG) (9).

### Plasma Cytokine and Chemokine Detection Assay

Neat plasma from Covid-19 and controls was used to measure 41 cytokines and chemokines using human Milliplex map cytokine assay kit (Millipore, Billerica, MA). The samples were acquired in a Bio-Plex 200 system (Bio-Rad, Hercules, CA) and cytokine concentrations were calculated using Bio-Plex manager software with a five-parameter (5PL) curve-fitting algorithm applied for standard curve calculation (10).

### Isotyping of Circulating Antibody From Blood

For analysis of the isotype composition of antibodies in circulation, plasma of T2DM, NDM patients, and healthy volunteers was analyzed by ProcartaPlex Human Antibody Isotyping Panels (Cat. No EPX070-10818-901, Invitrogen, Vienna, Austria), based on the manufacturer’s instructions. Briefly, antibody-coated magnetic bead mixtures were incubated with 25 μl of assay buffer, kit standards or diluted plasma (1:20000) samples in a ProcartaPlex 96-wells plate at room temperature for 1 h. Detection antibodies (25 μl) were then added, and the plates were incubated on an orbital shaker at 500 rpm for 30 min. Next, the wells were incubated with 50 μl of diluted Streptavidin-Phycoerythrin for 30 min. Plates were then washed using a hand-held magnetic plate washer. All incubations were performed at room temperature in the dark. Afterward, samples were suspended in 120 μl reading buffer and were acquired in a Bio-Plex 200 system (Bio-Rad, Hercules, CA) and cytokine concentrations were calculated using Bio-Plex manager software with a five-parameter curve-fitting algorithm (5PL) applied for standard curve calculation (11).

### Whole Blood Immunophenotyping by Mass Cytometry

For immunophenotyping, 270 μl of whole blood was collected, added to pre-coated Maxpar Direct Immune Profiling tubes, and incubated for 30 min at room temperature. For lysis, 250 μl of 1x BD FACs Lyse was added, followed by 10 min incubation. There were two consecutive washes with Maxpar water and cell staining buffer. The cells were then fixed with 4% formaldehyde and incubated for 10 min followed by washing at 800 g for 5 min. Eventually, the cells were suspended in 1 ml Iridium solution and stored in -80°C until acquisition. For acquisition, the cells were thawed, washed with cell staining buffer and cell acquisition solution. The cell density was adjusted to 1 million cells/ml in cell acquisition solution with 0.1% EQ Beads and acquired in a Helios Mass cytometer. FCS files were then normalized with CyTOF software V.7 (Fluidigm) and then exported and analyzed by FlowJo software V10.7 (BD Biosciences) (12, 13). All the procedures up to the formaldehyde fixation step were carried out inside the institute’s BSL-3 facility.

### Statistics

Statistical analysis was performed using the GraphPad Prism software, version 8.0.1. Data were presented as Mean ± Standard Deviation of Mean (SEM). Non-parametric Kruskal Wallis Test with *post hoc* Dunn’s multiple comparison test were used to compare the levels of cytokines, antibodies, and percentage of different immune cells among the three groups. Mann-Whitney U test was used to compare laboratory parameters between T2D and NDM patients. *P* values less than 0.05 were considered significant (*P < 0.05, **P < 0.01, ***P < 0.001, ****P < 0.0001).

### High Dimensional Analysis

For high dimensional analysis, t-SNE analysis was performed using FlowJo V.10.7 (BD Biosciences). The gating for analysis was done on live, intact, CD45^high^CD66b^low^ lymphocytes. Among a total of 100,000 events, 5000 events in the lymphocyte population were used per sample for this analysis. Five samples of T2D and healthy controls were used to compute t-SNE and a total of 25,000 events per group were concatenated and exported for t-SNE analysis. The default parameters in the software, iterations-1000, perplexity-30, eta-675, KNN algorithm-Exact (vantage point tree), gradient logarithm-Barnes-Hut were used to compute the t-SNE plot.

## Results

### Viral Load Does Not Corroborate With Inflammation in T2DM

**Figure 1:**
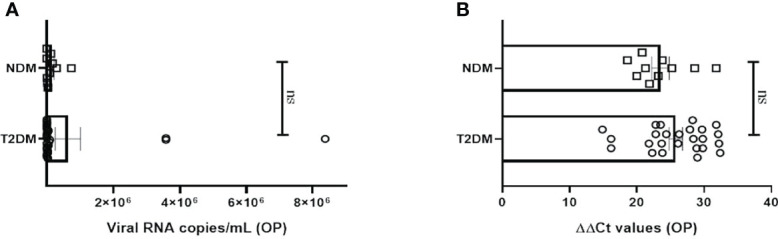
Analysis of viral load in oropharyngeal samples in Covid-19 patients. **(A)** Bar diagram depicting the viral copy number of oropharyngeal (OP) samples collected from T2DM (n = 25) and NDM (n = 10) COVID-19 patients. **(B)** Bar diagram representing the **ΔΔ** CT of OP samples of NDM and T2DM COVID-19 patients. The Mann-Whitney (non-parametric, two tailed) test was performed. ns, not significant. All error bars were SEM.

The study was executed on NDM and T2DM Covid-19 patients, who had been detected and admitted to a local tertiary COVID hospital. To examine for viral load, oropharyngeal (OP) samples were collected from various patients as per established protocol and processed in the BSL3 facility at ILS, Bhubaneswar. In most of the NDM and T2DM patients, there was no significant difference in the viral copy number or ΔΔ Ct values of OP samples at the time of sampling (**Figures 1A, B**). This suggests that the viral load in OP samples does not and did not correlate with disease severity in this population of the study. As the viral load present in the host did not dictate the severity of Covid-19 infection, we hypothesize that disease severity could have resulted as a consequence of altered metabolic status due to T2DM and chronic low-grade inflammation.

### Altered Cytokine and Chemokine Profiles in Covid-19 Patients With Type II Diabetes

**Figure 2:**
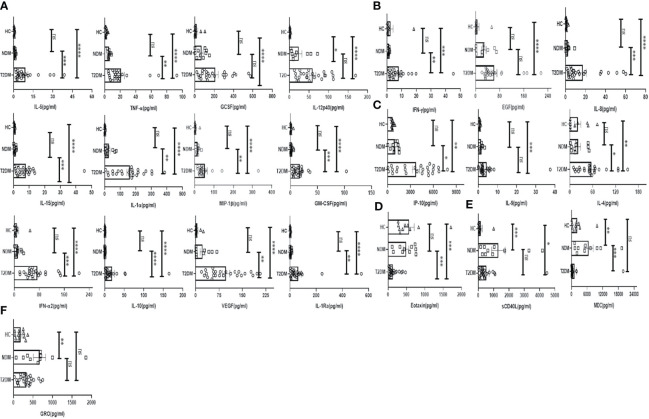
Analysis of the cytokines and chemokines in T2DM, NDM patients infected with SARS-CoV2. The bar diagrams represent cytokines and chemokines which were evaluated from COVID -19 positive plasma samples of T2DM (n = 25), NDM (n = 10), and Covid-19 negative and non-diabetic healthy controls (n = 10). **(A)** IL-6. TNF-α, GCSF, IL-12p40, IL-15, IL-1α, MIP-1β, GMCSF, IFN-α2, IL-10, VEGF, and IL-Rα were highly elevated in T2DM patients when compared with healthy controls and NDM patients. **(B)** IFN-γ, EGF, and IL-8 were moderately elevated in T2DM patients when compared to healthy controls and NDM. **(C)** IP-10, IL-9, and IL-4 were slightly elevated in T2DM patients when compared to healthy controls and NDM. **(D)** Eotaxin was moderately elevated in healthy controls and NDM when compared to T2DM patients. **(E)** sCD40L was elevated in both NDM and T2DM when compared to healthy controls, MDC was elevated in NDM patients when compared to T2DM patients. **(F)** GRO was elevated in NDM patients when compared to healthy controls. The Kruskal Wallis Test (non-parametric) with *post-hoc* Dunn’s multiple comparison test was performed. p < 0.05 was considered statistically significant (*), p < 0.01 was considered to be very significant (**), *P* < 0.001 was considered to be highly significant (***), *P* < 0.0001 was considered to be extremely significant (****), ns, not significant. All error bars were SEM.

To investigate the status of systemic inflammation concurrent with SARS-Cov2 infection, we did multiplexing analysis of 41 cytokines and chemokines from the plasma of T2DM and NDM patients to delineate the altered immune microenvironment in these patients. For comparison and for basal level expression of these cytokines and chemokines, we added 10 healthy volunteers and segregated the proteins into significant, moderate, and mild, based on the level of their expression in T2DM as compared to NDM and healthy volunteers. Accordingly, the levels of IL-6, TNF-α, G-CSF, GM-CSF, IFN-α2, IL-10, VEGF, IL-1Rα, IL-12p40, IL-15, IL-1α, and MIP-1β were found to be significantly elevated (p<0.0001, Kruskal Wallis Test), indicating the simultaneous release of both Type I and Type II cytokines in severity (**Figure 2A**). In contrast, there was moderate elevation in the levels of IFN-γ, EGF, IL-8, indicative of the ongoing antiviral reaction in these patients (**Figure 2B**). Among the slightly elevated ones, IP-10 (p=0.0365), IL-9, IL-4 (p=0.0146) showed higher significance (**Figure 2C**). Although IP-10 has been used as a real time marker for mortality in Covid-19 patients, we did not do a longitudinal patient tracking, and therefore cannot ascertain its role in mortality. In addition, IL-4 and IL-9 which are both type II cytokines have also been linked to severity of Covid-19 patients. Although our study could not find any significant difference in levels of type III signature cytokines such as IL-17 and IL-1β, the presence of type I and type II immune signatures were overlapping in multiple patients (data not shown). However, T2DM patients showed elevation of both type I and type II cytokines indicating a dysregulated immune response, prominent in these patients as earlier reported by Lucas et al. (**Figure 2**). As hypothesized above with near similar viral load in all patients, these results suggest that altered cytokine and chemokine profiles could be a result of dysregulated immune status and altered metabolic status as previously mentioned.

Interestingly, the levels of chemokines as opposed to cytokines were higher in NDM and healthy volunteers as opposed to T2DM patients. For example, the levels of Eotaxin were found to be lower in T2DM than in NDM patients and healthy controls (**Figure 2D**). In general, Eotaxin is associated with chemoattraction of eosinophils, basophils, and neutrophils. Previous studies have reported its increase with severity but in our study, the population indicated a reverse trend; decreasing Eotaxin levels in the T2DM patients. This can be indicative of an impaired healing process as previous reports have suggested a positive correlation between increasing number of eosinophils and healing. In addition, sCD40L was elevated in NDM patients as compared to T2DM and healthy volunteers (**Figure 2E**). Since sCD40L has been associated with immunosuppression, it is indicative of an anti-inflammatory immune response in NDM patients, which is clearly absent in T2DM cases. Similarly, the levels of MDC (macrophage derived chemokine) were also higher in NDM as compared to T2DM patients, demonstrating its potential role in immune suppression (**Figure 2E**). In addition, GRO levels were higher in both NDM and T2DM patients as compared to healthy controls (**Figure 2F**). As GRO serves as a chemoattractant for neutrophils, it is indirectly indicative of the status of underlying inflammation in NDM and T2DM patients. Collectively, our cytokine and chemokine profile of T2DM patients compared to NDM and controls showed an increasing trend in the inflammatory cytokine panel and augmented levels of chemokines that were primarily responsible for repair and angiogenesis indicating toward the inadequate inflammatory response required for viral clearance but significantly better tissue repair mechanisms in place.

### Circulating Antibody Isotype Profiling Showed a Skewed Inflammatory Profile in Covid-19 Patients With Type II Diabetes

**Figure 3:**
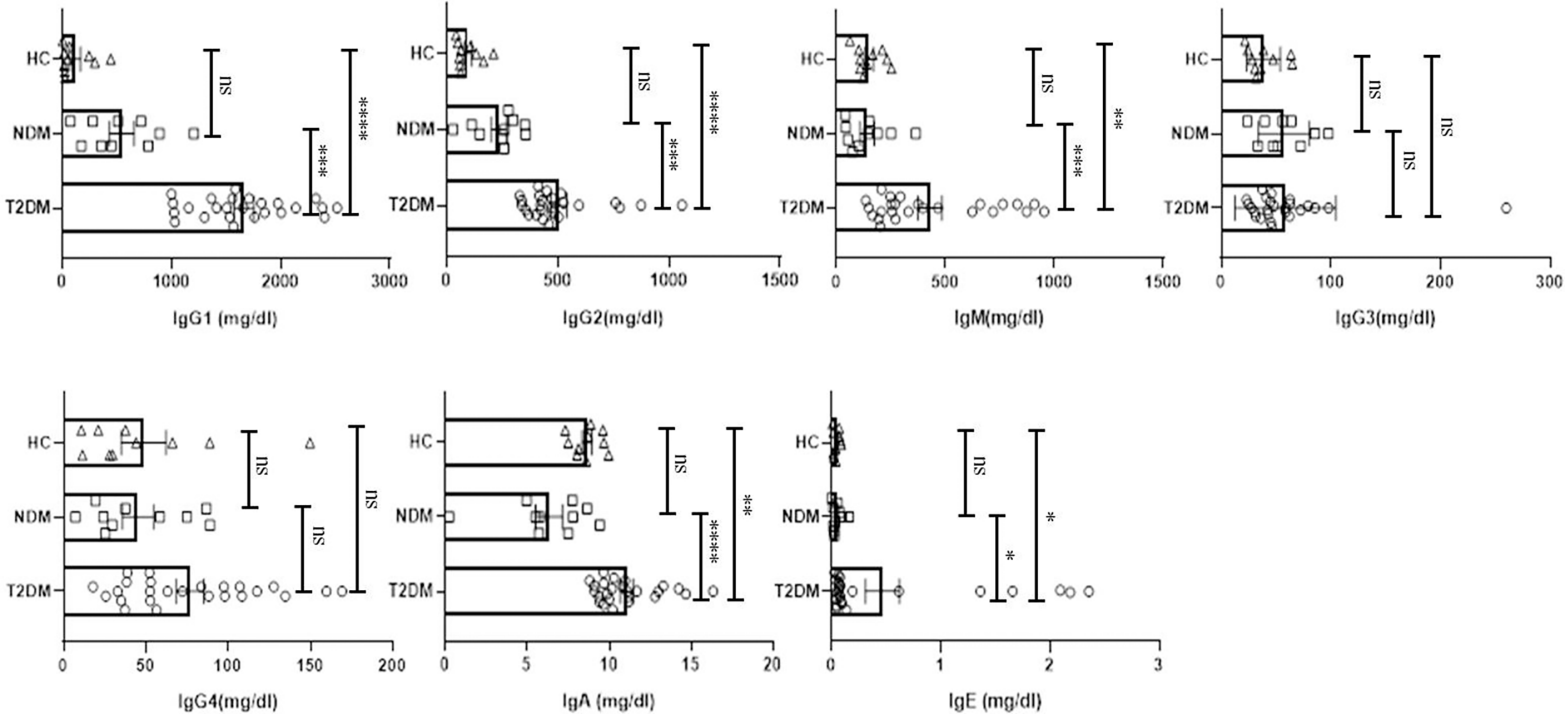
Analysis of the circulating antibodies in T2DM, NDM patients infected with SARS-CoV2. The bar diagram representing circulating antibody isotypes (IgA, IgM, IgG1, IgG2, IgG3, IgG4, and IgE) which were evaluated from COVID -19 positive plasma samples of T2DM (n = 25), NDM (n = 10), and Covid-19 negative and non-diabetic healthy controls (n = 10). The Kruskal Wallis Test (non-parametric) with *post-hoc* Dunn’s multiple comparison test was performed. p < 0.05 was considered to be statistically significant (*), p < 0.01 was considered to be very significant (**), *P* < 0.001 was considered to be highly significant (***), *P* < 0.0001 was considered to be extremely significant (****), ns, not significant. All error bars were SEM.

Various antibody isotypes correlate with the clinical phenotype of an infection and is therefore an important indicator of the type of immune response. However, altered immune status including immune pathologies and deficiencies, etc. skew antibody profiles because of cytokine bias that is not physiological. Since cytokine *milieu* in T2DM and NDM patients was skewed, we wanted to define the circulating antibody isotypes in these patients as opposed to healthy controls. We did Luminex-based multiplex assay from their plasma and quantified the amount of IgA, IgM, IgE, IgG1, IgG2, IgG3, and IgG4. Here, we found IgG1 and IgG2 to be significantly elevated in T2DM patients as compared to healthy controls (p=<0.0001) and NDM patients (p=0.0024 for IgG1 and p=0.0033 for IgG2), correlating with the higher levels of type I cytokines in these patients. IgE, being a hallmark of type II immune response, was also elevated in T2DM as compared to NDM (p=0.0453) and healthy control (p=0.0347). Alongside, IgA being associated with mucosal immunity was also higher in T2DM patients as compared to NDM (p=0.0012) and healthy controls (p=0.0140). As IgM is the first antibody isotype to appear during the course of infection, accordingly we found its levels to be higher in T2DM patients as compared to NDM (p=0.0178) and healthy controls (p=0.0017) (**Figure 3**). In summary, the antibody isotype profile was concurrent with the cytokine levels, thus confirming and furthering the status of disease severity in Covid-19 patients. However, these antibody isotypes and levels suggest being counterproductive as they seem to be incapable of either consolidating anti-viral responses or even being protective against viral migration in the host.

### Dysregulated Innate Immune System in Covid-19 Patients With Type II Diabetes

**Figure 4:**
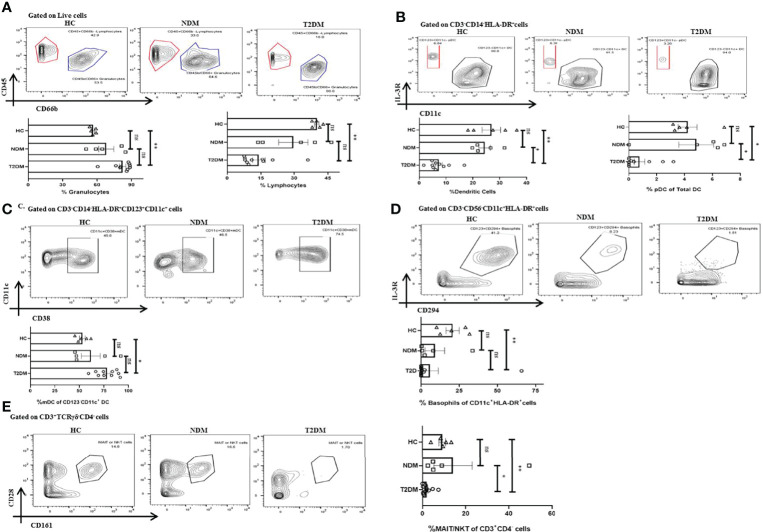
Innate immune cells in T2DM and NDM patients. Whole blood from Covid-19 positive T2DM (n = 10), NDM (n = 5), and Covid-19 negative and non-diabetic healthy controls (n = 5) was stained heavy metal tagged antibody and analyzed in a mass cytometer. **(A)** Representative gating strategy to identify CD45^+^CD66b^low^ lymphocytes and CD45^-^CD66b^high^ granulocytes. Statistical analysis of percentage of granulocytes and lymphocytes. **(B)** Gating strategy for CD123^+^CD11c ^–^ pDCs. Statistical analysis for the frequency of total DCs and pDCs. **(C)** Gating strategy for CD11c^+^CD38^+^ mDCs. Statistical analysis for the percentage of mDCs. **(D)** Gating strategy for CD123^+^CD294^+^ basophils. Statistical analysis for the frequency of basophils. **(E)** Gating strategy for MAIT/NKT cells. Statistical analysis for the frequency of MAIT/NKT cells.The Kruskal Wallis Test (non-parametric) with post-hoc Dunn’s multiple comparison test was performed. p < 0.05 was considered statistically significant (*), p < 0.01 was considered to be very significant (**), ns, not significant. All error bars were SEM.

With the cytokine multiplexing and antibody isotyping indicating an altered immune response, our next objective was to delineate the alteration in the cells of innate and adaptive immune system during Covid-19. Based on the surface markers analyzed, we found T2DM patients showing significant increase in *CD45^low^CD66b^high^* granulocytes as compared to healthy controls (p=0.0074), which is concurrent with the increase in the levels of IL-8 (**Figure 4A**). The bulk population of these granulocytes were neutrophils, though we did not find any significant difference between the levels of neutrophils in T2DM, NDM, and healthy controls (data not shown). Also, dendritic cell populations were variable, with their numbers decreasing in T2DM as compared to NDM and healthy controls (**Figure 4B**). This contrasted with the increase in IFN-α2 levels, indicating toward dysregulated dendritic cell population in T2DM patients (**Figure 2A**). Within the dendritic cell population, plasmacytoid dendritic cells (pDCs) decreased in T2DM patients as compared to NDM and healthy controls (**Figure 4B**). However, percentage of myeloid dendritic cells (mDCs) increased in T2DM patients as compared to NDM and healthy controls (**Figure 4C**). This showed a significant depreciation in T2DM patients’ capability to upregulate anti-viral response while increased mDC with a Th2 bias is clearly detrimental to antiviral causes. The percentage of basophils was also reduced in T2DM patients as compared to healthy volunteers, which correlated with the decrease in levels of chemokine, Eotaxin (CCL11) (**Figure 4D**). Although we did not observe any variation between T2DM, NDM, and healthy controls in the populations of gamma delta cells and natural killer cells, there was reduction in the percentage of MAIT/NKT cells in T2DM patients as compared to healthy and NDM patients (**Figure 4E**). As reported earlier (data not shown), there were no changes in the overall or subsets of monocytes and natural killer cells. Altogether, alterations in the innate immune cell population are suggestive of an aberrant immune response incapable of controlling the infection.

### Heterogenicity in Lymphocyte Population in Covid-19 Patients With T2DM, NDM, and Healthy Controls

**Figure 5:**
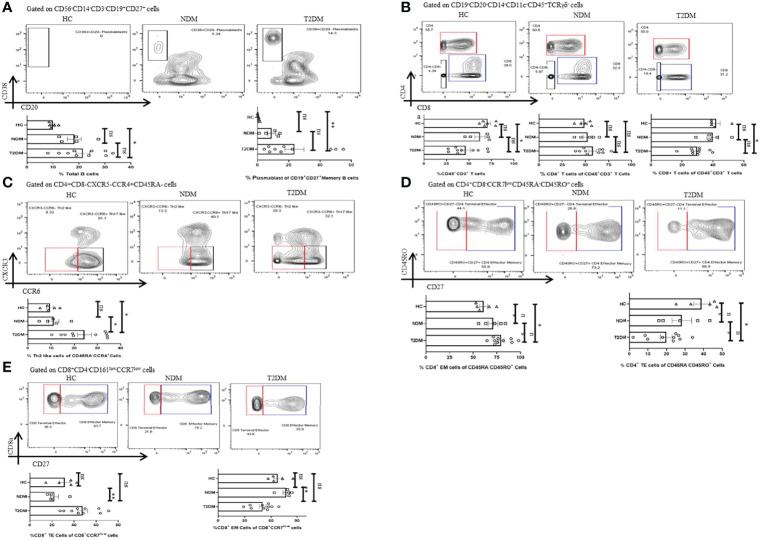
Adaptive immune cells in T2DM and NDM patients. Whole blood from Covid-19 positive T2DM (n = 10), NDM (n = 5), and Covid-19 negative and non-diabetic healthy controls (n = 5) was stained heavy metal tagged antibody and analyzed in a mass cytometer. **(A)** Representative gating strategy to identify CD38^+^CD20 ^–^ plasmasblasts. Statistical analysis of percentage of plasmasblasts. **(B)** Gating strategy to identify CD45^+^CD3^+^CD4^+^ and CD45^+^CD3^+^CD8^+^ T cells. Statistical analysis for the frequency of CD3^+^, CD4^+^, and CD8^+^ T cells. **(C)** Gating strategy for CD4^+^CXCR3^-^CCR6^-^Th2 like cells. Statistical analysis for the percentage of Th2 like cells. **(D)** Gating strategy to identify CD4^+^ TE(CD45RO^+^CD27^-^) and CD4^+^ EM cells (CD45RO+CD27^+^). Statistical analysis for the percentages of CD4^+^ TE and EM cells. **(E)** Gating strategy to identify CD8^+^ TE (CCR7 ^–^ CD27^-^) and EM(CCR7^-^CD27^+^) cells. Statistical analysis for the percentages of the CD8^+^TE and EM cells. The Kruskal Wallis Test (non-parametric) with *post-hoc* Dunn’s multiple comparison test was performed. p < 0.05 was considered to be statistically significant (*), p < 0.01 was considered to be very significant (**), ns, not significant. All error bars were SEM.

Lymphocytes are instrumental in fighting infections and earlier reports indicated toward its decreasing trend in T2DM patients. There was a moderate decrease in the percentage of lymphocytes in T2DM as compared to the healthy controls (**Figure 4A**). Among lymphocytes, the percentage of total B cell population expanded in T2DM patients as compared to healthy controls (**Figure 5A**) and was in direct correlation with increased IgG1 and IgG2 in the former (**Figure 3**). Further analyses revealed a significant increase in plasmablast population in T2DM patients as compared to healthy controls. Additionally, we found a significant decrease in the percentage of CD3^+^ T cells in T2DM patients as compared to healthy controls suggesting lymphopenia associated with increased severity (**Figure 5B**). Within the T cell population, there was an increase in the percentage of CD4^+^ T cells (statistically insignificant) but a decrease of CD8^+^ T cells (**Figure 5B**). Further, in-depth analysis of CD4^+^ T cells revealed that T2DM patients had an increase of the Th2 subtype as compared to NDM or healthy volunteers (**Figure 5C**). This can be correlated with the increased levels of IL-4 and IL-9, found in the plasma of these patients (**Figure 2C**) and is generally unproductive in protecting against viral infections. However, Th1, Th17, Treg, and Tfh cells did not show any statistically significant difference, which can be attributed to the time of sampling or be a consequence of dysregulated immune response. There was also no significant difference in naïve CD4^+^ T cells or central memory CD4^+^ T cells within the groups but terminal effector (TE) cells were decreased, and effector memory (EM) cells were increased in T2DM patients as compared to healthy controls (**Figure 5D**). This contrasted with the CD8^+^ compartment, where TE cells were increased in T2DM patients as compared to NDM but there was no difference between T2DM and healthy controls suggesting a robust response in NDM patients as compared to T2DM and a certain degree of cross-reactive reaction occurring in these T2DM patients (**Figure 5E**). Similarly, there was a significant decrease of EM cells in T2DM as compared to NDM while no difference was observed in healthy controls when compared to T2DM or NDM. Collectively, a bias was found toward terminal effector CD8^+^ T cells in T cell immune response which was suggestive of an effective ongoing anti-viral response.

### High Dimensional Analysis Reveals Immune Perturbations in T2DM Patients

**Figure 6:**
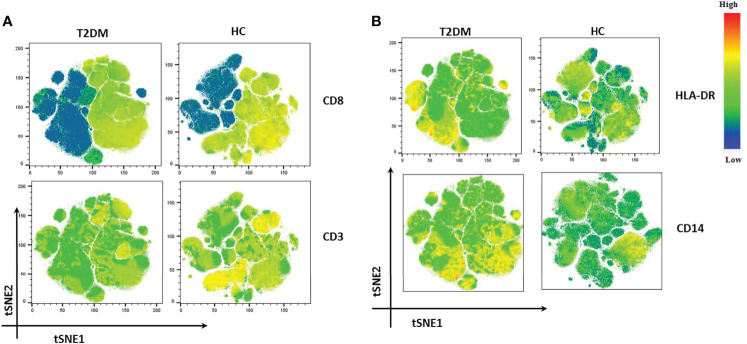
t-SNE analysis of lowest and highest expressed markers in T2DM patients. **(A)** CD8 and CD3 showing decreased expression in T2DM patients. **(B)** HLA-DR and CD 14 showing increased expression in T2DM patients when compared to controls.

We also performed t-distribution stochastic neighbor embedding (t-SNE) analysis to understand how different markers reported previously show variability in Covid-19 patients in our population of study. t-SNE analysis revealed a decrease in the expression of CD3 and CD8 in T2DM patients as compared to healthy volunteers (**Figure 6A**). This was clearly suggestive of a poor anti-viral response. On the contrary, there was increase in the expression of CD14, CD38, and HLA-DR in T2DM patients as compared to healthy volunteers (**Figure 6B**) indicative of the ongoing inflammation in the T2DM patients. Taken together, our data indicates a compromised antiviral immunity and low-grade inflammation that is counterproductive to infection control.

## Discussion

The Covid-19 pandemic has brought an unprecedented global devastation to the lives and livelihood of the common people, a breakdown of the best healthcare system, and economic collapse across the globe. Until March 2022, India reported approximately 43 million cases, being second in place to the United States. Unfortunately, a bulk population of Covid-19 cases in India who had succumbed to the infection had co-morbidities and among these, type II diabetes was the most common. At present, there are no significant reports from India providing an insight into the variations in immune response, extent of inflammation, and outcome in T2DM patients as compared to NDM patients and healthy volunteers. In this study, we attempted to describe and compare immune response, viral loads, and clinical parameters among T2DM, NDM, and healthy volunteers. To that end, 25 patients with T2DM, 10 NDM patients, and 10 healthy volunteers were studied for cytokine, chemokine, viral loads, clinical parameters, and antibody isotyping assays for understanding the immunopathology associated with the virus. 10 T2DM, 5 NDM, and 5 healthy volunteers were studied for whole blood mass cytometry assay to determine the difference in immune cells among the groups. While it is understood that this is a very small group, some clear patterns are established here suggesting that the chronic meta inflammation in type II diabetes might play a role in further precipitating the inflammatory signatures in Covid-19.

It is known that mild and controlled inflammation serves as one of the major initiator mechanisms of the immune system that is extremely crucial in fighting infection (14). However, unregulated upregulation of circulatory cytokines and dysregulated inflammatory responses that eventually lead to a cytokine storm have been evinced in Covid-19 infection (2, 4). Additionally, previous reports suggest that patients with T2DM have a higher vulnerability for SARS-CoV2 infection (15, 16) where persistent low-grade inflammation associated with T2DM was evinced but not co-related to be causal. Our data taken together with biochemical, cytokine, and antibody profile strongly indicate that chronic low-grade inflammation and the consequential dysregulated immune response might be responsible for the upsurge of inflammatory responses in Covid-19 patients leading to severe infection, organ damage, and mortality.

Our preliminary objective in the study was to assess the differences in viremia in oropharyngeal swab between the two groups. However, viral load of OP samples did not show any significant difference between the two groups. This was in accordance with previous studies, where it was observed that the viral load in nasal and throat swab samples was similar in the NDM and T2DM patients (17, 18). Here, the T2DM are considered as severe Covid-19 patients, based on the severity of their symptoms. In addition, the NDM patients either had mild Covid-19 symptoms (n=4) or were asymptomatic (n=6). This is evident from the clinical parameters as T2DM patients had higher levels of CRP which is a non-specific inflammatory marker, dysregulated glucose metabolism as evident from higher plasma glucose, glycated hemoglobin, and impaired blood coagulation as interpreted from higher d-dimer levels (19–22). Our viremia and biochemical data strongly suggest that the same or even similar infection loads have an adverse effect on already altered biochemical status and the resultant response culminates in non-effective viral clearance, organ damage, and even mortality. On the other hand, viremia and immunological co-relation suggests that altered immune cells, non-productive, non-protective, and counterproductive immune responses through cytokines and chemokines could be the mediators.

Cytokines and chemokines are soluble mediators that control the migration of immune cells to the site of inflammation, providing a pro – or anti-inflammatory environment which essentially shapes the type of immune response during a disease. In our T2DM patients who were infected with SARS-CoV2, higher expression of both type I and type II cytokines was evinced. Previous reports suggest the involvement of both types of cytokines in aggravating the severity of the disease. Increase in type I cytokines such as IFN-γ ; TNF-α, IFN-α2, IL-6, GMCSF, and IL-8 indicated a robust antiviral and inflammatory response occurring in these T2DM patients. Additionally, these patients also showed an increase in type II cytokines such as IL-4 and IL-9, representing Th2 cells and some anti-inflammatory response. Here, we understand the presence of both inflammatory and anti-inflammatory cytokines in T2DM patients as indicators of a dysregulated immune response where the anti-inflammatory response was clearly incapable of shutting down the cytokine storm in these patients (23). Additionally, we did not find any significant difference in inflammatory cytokines between NDM patients and healthy controls suggesting that the immune homeostasis is in place already. This may be because inflammation was mild and most of the patients were asymptomatic in this group, corroborating with similar findings from previous studies (24, 25). However, in diabetic patients, there is an increase in the inflammatory cytokines, significant CRP levels, and its sustained increase and the presence of blood clots. As similar symptoms are associated with Covid-19, i.e., elevated CRP levels, D-dimer, and inflammatory cytokines, this suggests a synergistic effect found in T2DM patients infected with SARS-Cov2, which was reduced or absent in NDM patients. Additionally, our study suggested an increase in IL-15 and 1L-7 in T2DM patients when compared to NDM and healthy volunteers. As it is known that IL-15 is crucial for promoting cytotoxic activity of both NK and CD8^+^ T cells, and is also involved in memory CD8^+^ T cell differentiation, it could have led to increase in the CD8^+^ population (26). However, we found a decrease in the CD8^+^ cell population, specifically in the EM compartment, again indicating a dysregulated or non-responsive immune response in these patients. This might be a consequence of reduced receptors for IL-15 in these cells. As IL-7 is also required for T cell development and maintenance (27), its increased numbers in T2DM patients partially corroborated with the increase in CD8^+^ TE cells and CD4^+^ EM cells. However, because of immune dysregulation, neither the elevated levels of the above-mentioned cytokines nor the CD8^+^ T cells are capable of controlling the inflammation.

The sCD40L is one of the major co-stimulatory molecules on activated T cells that interact with CD40 on B cells and is responsible for immunoglobulin isotype switching in the membrane-bound form (28). In our study, levels of sCD40L were elevated in NDM as compared to the T2DM patients indicating that the NDM patients may be undergoing an immunosuppressive reaction, as elevated sCD40L is also associated with immunosuppression. GRO (CXCL1) is a chemokine that attracts a variety of immune cells, particularly neutrophils, and is also implicated in the wound healing process (29). Similar to sCD40L levels, GRO levels were also higher in NDM as compared to T2DM patients indicating that both inflammation and healing were occurring simultaneously in these patients. However, the healing mechanism was poorer in T2DM patients. The levels of macrophage-derived chemokine (MDC/CCL2) were also higher in NDM patients than T2DM patients. As MDC is known to be elevated in lung inflammation and hemorrhage but reduced in T2DM symptomatic patients, this also suggests a possible immunosuppressive function of MDC in this population (30).

To understand how different antibody isotypes influence the clinical phenotype in T2DM and NDM patients, we did an antibody isotyping from the plasma of these patients. Here, we found significant differences in the levels of IgA, IgM, IgG1, IgG2, and IgE between the two groups but IgG3 and IgG4 did not show any significant differences and we understand that the structure and function of different antibody isotypes vary from one another. For example, IgG1, IgG2, and IgG3 can fix complement (31). However, IgG4 cannot fix complement and is unable to induce antibody mediated cell cytotoxicity (ADCC). Similar to previous reports, we found an increase in the levels of IgE in T2DM patients (8, 16). This corroborated with the increase in the Th2 subset along with increase in cytokines, IL-4 and IL-9 in T2DM patients, as it is known that IgE is associated with Type II response and is generally not productive or protective in anti-viral responses. In addition, there was also an increase in IgG1 and IgG2 in these patients, indicative of the inflammatory response. However, as IgE was high in these T2DM patients, we could see no significant difference in IgG4 levels. This could be accredited to the competition between IgE and IgG4 against each other, for fixation sites in basophils and mast cells, as suggested by previous reports (32) and IgG4 is important in dictating anti-inflammatory response. IgA levels were also found to be higher in T2DM, indicating a viral immune response at mucosal surfaces (33). With respect to IgM, its increased levels indicate an ongoing infection and at least partial protection. However, the lack of any longitudinal profiling restricts us from understanding its specificity to antigen (34).

With respect to our whole blood analysis by mass cytometry, we found an increase in the percentage of granulocytes in T2DM patients when compared to healthy controls indicating underlying inflammatory responses in these patients. This is consistent with other reports which suggested an increase in granulocytes with increasing severity (15, 35). On the other hand, we found a decreasing percentage of monocytes in T2DM patients as compared to NDM patients (**Table 1**), although the difference was not significant. This has also been reported in a previous study showing the absence of any significant changes in the total monocytes and its subsets in T2DM patients with various comorbidities, T2DM being one of them (15). Additionally, we also found that T2DM patients showed a decreasing trend in lymphocyte percentage as compared to NDM and healthy volunteers, as reported in previous studies (4, 8, 15). One of the significant findings from our study is that there is a drastic reduction in the percentage of total dendritic cells in T2DM patients when compared to both NDM and healthy controls. Within the dendritic cell compartments, there is a decrease of pDCs but mDCs have increased in T2DM patients when compared with healthy controls. In general, pDCs are known to secrete type I interferons in response to viral infection (36). However, our study has shown a decrease in the percentage of pDCs but an increase in the cytokine IFN-α2. This suggests that although these cells are secreting high levels of type I interferon, their numbers are depleted in T2DM patients and, thus, are unable to control the infection. With respect to mDCs, they are known to secrete the cytokines IL-12 and TNF-α, which polarizes the T cell toward a type I response, crucial for controlling the viruses (37, 38). Our study also observed an increase in the population of mDCs along with an increase in the cytokines, IL-12p40, TNF-α in T2DM. Although certain reports have suggested a decrease of mDCs along with pDCs, we observed the presence of high numbers of mDCs in diabetic patients, indicating an ongoing low-grade inflammation, which is further augmented with Covid-19 infection. However, since we did not do a longitudinal profiling, we could not delineate the mDC dynamics in these patients (39, 40).

With respect to basophils, the literature suggests a decrease in its population with increasing severity (41). We found a similar trend, as the basophil population decreased in T2DM patients as compared to healthy controls. Similarly, MAIT/iNKT cells were significantly decreased in T2DM patients as compared to NDM and healthy controls. However, no significant differences were observed in the percentage of gamma delta T cells. A decrease in MAIT/iNKT could indicate a migration to other inflamed tissues including the lungs, as suggested by the previous reports. With respect to NK cell population, it is known that they play a crucial role in fighting viral infections and previous reports suggested a decrease in their numbers in T2DM cases. However, our population of study did not show any significant difference among the groups and as mentioned earlier may be a consequence of the time of sampling of patients (42, 43).

In the case of B cells, we observed a slight elevation in its percentage in T2DM patients, when compared to NDM and healthy controls, but statistical difference was found only between T2DM patients and healthy volunteers. Although no difference was observed in naive or memory B cell compartment, plasmablasts were significantly elevated in T2DM patients as compared to healthy controls. The increase in plasmablasts in T2DM Covid-19 has also been reported in other studies (8, 15) but unlike our study, they could not correlate it with any co-morbidity. Interestingly, there are reports suggesting an increase in extrafollicular B cell response in Covid-19 and other inflammatory diseases such as systemic lupus erythematous. Since most of the Covid-19 patients had type II diabetes in our study, we hypothesize that these two factors might have augmented the plasmablast production. Although we did not analyze the clonality of B cells, an earlier study suggested an oligoclonal expansion of B cells in T2DM Covid-19, in turn correlating with increased plasmablast production. Increased B cell population in T2DM patients also corroborated with the fact that most of the immunoglobulins (IgM, IgA, IgG, and IgE) were elevated in these patients as opposed to NDM and healthy volunteers. Altogether, we found an increase in the plasmablast population in T2DM as compared to NDM patients and healthy volunteers, suggesting that the low-grade inflammation in diabetic patients resulted in an increase in the plasmablast population, which was augmented further with Covid-19 infection in these patients. Altogether, these findings suggested that elevation of plasmablasts and various antibody isotypes was incapable and inadequate in providing protection to the host and might in turn be responsible for immune complexes targeting the organs.

T2DM patients also displayed a lower percentage of CD45^+^CD3^+^ T cells as compared to healthy controls. Within the CD3^+^ T cell population, there was a decrease in the percentage of CD8^+^ T cells in T2DM as compared to healthy volunteers. However, CD4^+^ T cells did not show any statistically significant difference among the groups. Earlier reports have suggested the loss of CD8^+^ T cells being greater than CD4^+^ T cells. Since our population of study was small with heterogenous manifestation, this could explain why CD4^+^ T cells did not show any difference among the groups. Additionally, several reports indicate towards T cell apoptosis or migration to other tissues in SAR-CoV2 infection, leading to decrease in T cell population in the periphery and could account for the decrease in T cells in the T2DM patients (44–46). In general, there was decrease in the CD3^+^ T cell population, where the CD8^+^ compartment was affected more significantly than CD4^+^. The above observation indicated the inflammatory environment together with the SARS-CoV2 infection, is causing activation induced cell death in T cells particularly of the CD8^+^ cells and compromising antiviral response.

Type II diabetes has been reported to have various aberrancies such as an impaired differential potential and secretion of multiple proinflammatory cytokines such as TNF-α and IFN-γ (39, 47). Although we could not find any significant difference among the groups in the percentage of Th1 like, Th17 like, Treg, or cTfh subtypes, there was an increase in the pro-inflammatory cytokines. We understand that the absence of difference among the groups with respect to T cell subsets can be accounted to the time of sampling. On the other hand, our study found an increase in the percentage of Th2 cells secreting type II cytokines such as IL-4 and IL-9. The high numbers of Th2 cells partially explained the augmented levels of immunoglobulins in these patients, specifically IgE which is induced by cytokines such as IL-4. Taken together, this indicated that the diabetic patients already had low grade inflammation. When encountered with Covid-19, dysregulation in the immune system escalated to such levels that even with the increase in Th2 population secreting anti-inflammatory cytokines, inflammation could not be subdued. The presence of Th2 type response in the above T2DM patients indicates a dysregulated suppression and also an improper antiviral response.

We also analyzed for the naïve, effector, and memory compartments in both CD4^+^ and CD8^+^ T cells. In CD4^+^ T cells, we did not see any difference in the overall percentage of naive (CCR7^+^CD45RA^+^CD45RO^-^) and central memory cells (CCR7^+^CD45RA^-^CD45RO^+)^ among the various groups. However, effector memory cells (CCR7^-^CD45RA^-^CD45RO^+^CD27^+^) were elevated in T2DM patients when compared to healthy controls, suggesting cross-reactivity with other families of coronavirus, as reported by a previous study from India (48, 49). An important point to note here is that central memory cells did not show any significant difference, and this may be due to the fact that CM cells are lymph node residents as opposed to EM cells, which circulate in the blood and, thus, respond faster to the antigen (data not shown). However, since we did not analyze for the antigen specific cells, therefore we were unable to find any significant difference in the percentage of CM cells. Terminal effector (CCR7^-^CD45RA^-^CD45RO^+^CD27^-^) cells were decreased in T2DM patients with Covid-19 as compared to healthy controls, corroborating with the observation that these cells are first to react with the virus and the subsequent pro-inflammatory cytokine *milieu* is responsible for the apoptosis of these cells.

In CD8^+^ T cell compartment, EM (CCR7^-^CD27^+^) cells were elevated in NDM as compared to T2DM patients, indicating an effective immune response in these patients but less effective in T2DM patients. TE (CCR7^-^CD27^-^) cells increased in T2DM patients indicating that these cells are highly inflammatory and might be insensitive to AICD (49–51).

Altogether, our data suggests that meta inflammation present in these T2DM patients is responsible for unproductive and unprotective anti-viral response while also resulting in aggravation of the inflammation occurring due to Covid-19. With T2DM being one of the most common morbidities present in India, these patients remain most vulnerable and are susceptible to secondary infections like mucormycosis. Age also has a profound effect on SARS-CoV2 severity but in our study, we did not find any significant variation between ages of NDM and T2DM group, median age of the NDM group being 49 and the T2DM group being 46. This observation is in line with another study that shows in low – and middle-income countries infection and death rate are highest among the age group 55 and lower (52). Though vaccinations are lowering the severity of the disease, reported waning antibody response in 3-6 months is a major concern apart from how long the protective immunity from T cells will work in these patients has also yet to be ascertained. Our study is essentially aimed at understanding how a low grade chronic inflammatory disorder such as type II diabetes dictates the immune response during the pathogenesis of Covid-19. We understand that our study is not devoid of limitations such as a small sample size, unavailability of T2DM samples without SARS-CoV2 infection during the course of our study, absence of longitudinal study or follow up, but nevertheless it will help in understanding the vulnerability of these patients and subsequent planning of vaccine coverage in these patients or future vaccine booster dosages to them.

## Data Availability Statement

The original contributions presented in the study are included in the article/**Supplementary Material**. Further inquiries can be directed to the corresponding authors.

## Ethics Statement

The studies involving human participants were reviewed and approved by the Institutional Human Ethics Committee, Institute of Life Sciences. The Institutional Ethics Committee (IEC)/Institutional Review Board (IRB) reference number is 106/HEC/2021. Written informed consent to participate in this study was provided by the participants’ spouse/next of kin.

## Author Contributions

Experimental design and conceptualization – SD, SS, and GB. Sample collection and processing – RS, PP, SaS, SS, GB, SaC, PB, and PN. Mass cytometry experiments – SS, GB, and SKS. Chemokine and cytokine multiplexing – SS, SaC, and GB. Antibody isotyping – SS, SaC, and AD. RT-PCR and virological assays – SaC, AD, and SC. Data analysis – SS, GB, SaC, AD, SC, and SD. Manuscript drafting – SS, GB, SaC, SC, and SD. Manuscript editing and review – GB, SS, RS, PP, AP, SC, and SD. All authors contributed to the article and approved the submitted version.

## Funding

This study was supported by the core funding of Institute of Life Sciences, Bhubaneswar, Department of Biotechnology, India. SS was funded by the DBT fellowship. GB, SaC, and SKS were funded by the CSIR fellowship.

## Conflict of Interest

The authors declare that the research was conducted in the absence of any commercial or financial relationships that could be construed as a potential conflict of interest.

## Publisher’s Note

All claims expressed in this article are solely those of the authors and do not necessarily represent those of their affiliated organizations, or those of the publisher, the editors and the reviewers. Any product that may be evaluated in this article, or claim that may be made by its manufacturer, is not guaranteed or endorsed by the publisher.
