# Supplementary material for: Underlying Co-Morbidity Reveals Unique Immune Signatures in Type II Diabetes Patients Infected With SARS-CoV2
Source: Front Immunol. 2022 Apr 27;13:848335. doi: 10.3389/fimmu.2022.848335 (PMC9094480; doi:10.3389/fimmu.2022.848335)
Supplement: Supplementary file 1 [file Table_1.docx]

|  | HEALTHY CONTROLS(HC), n=10 |
| --- | --- |
| Age, median (IQR), years | 33(30-35.75) |
| Sex, male/female, n (%) | 6(60%)/4(40%) |
| Platelets 10^3^/µL median (IQR) | 239(200.5-260) |
| White Blood Cells 10^3^/µL, median (IQR) | 6.49(5.96-7.32) |
| Neutrophils %, median (IQR) | 56.75(49.45-63.5) |
| Lymphocytes%, median (IQR) | 32.9(30.43-35) |
| Eosinophils %, median (IQR) | 2.4(1.9-3.2) |
| Monocytes%, median (IQR) | 1.55(1.05-1.68) |
| Haemoglobin, gm/dl median (IQR) | 12.5(11.75-14.30) |

**ST1. Demography and Haematological parameters of Healthy Controls**
