# Supplementary material for: Underlying Co-Morbidity Reveals Unique Immune Signatures in Type II Diabetes Patients Infected With SARS-CoV2
Source: Front Immunol. 2022 Apr 27;13:848335. doi: 10.3389/fimmu.2022.848335 (PMC9094480; doi:10.3389/fimmu.2022.848335)
Supplement: Supplementary file 2 [file Table_2.docx]

| **REAGENT** | **CATALOGUE NUMBER** |
| --- | --- |
| **MILLIPLEX MAP Human Cytokine/Chemokine Magnetic Bead Panel - Immunology Multiplex Assay** | **Millipore -HCYTOMAG-60K** |
| **Antibody Isotyping 7-Plex Human ProcartaPlex™ Panel** | **Invitrogen- EPX070-10818-901** |
| **BD FACS ^TM^ FACS Lysing Solution** | **BD Biosciences-349202** |
| **Maxpar® Direct™ Immune Profiling Assay™** | **Fluidigm- SKU 201325** |
| **Human TruStain FcX™ (Fc Receptor Blocking Solution)** | **Biolegend-422302** |
| **Formaldehyde Solution** | **Qualigens-12755** |
| **TRUPCR SARS-CoV-2 RT qPCR Kit V-2.0.** | **3B BlackBio Biotech -** **3B304** |

**ST2. Reagents used in the study**
