# Supplementary material for: Underlying Co-Morbidity Reveals Unique Immune Signatures in Type II Diabetes Patients Infected With SARS-CoV2
Source: Front Immunol. 2022 Apr 27;13:848335. doi: 10.3389/fimmu.2022.848335 (PMC9094480; doi:10.3389/fimmu.2022.848335)
Supplement: Supplementary file 3 [file Table_3.docx]

| **Drugs administered** | **T2DM**  n=25 | **NDM**  n=10 |
| --- | --- | --- |
| Pantoprazole, n (%) | 25/25 (100 %) | 10/10 (100%) |
| Ivermectin, n (%) | 25/25(100%) | 10/10(100%) |
| Azithromycin, n (%) | 25/25(100%) | 5/10 (50%) |
| Ascorbic acid, n (%) | 25/25 (100%) | 10/10(100%) |
| Multivitamin with Zinc, n(%) | 25/25 (100%) | 10/10(100%) |

**ST3. Details of Drugs administered to T2DM and NDM patients infected with SARS-CoV2**
